# Mimicking Mother Nature in the Field of Human Reproduction?

**DOI:** 10.1055/s-0043-1768460

**Published:** 2023-04-27

**Authors:** Jesús Alfredo Berdugo Gutierrez, Omar Ammar, Stefan Du Plessis, Walter Cardona Maya

**Affiliations:** 1 Universidad Nacional de Colombia, Sede Orinoquia, Arauca, Colombia; 2 Reproduction Group, Department of Microbiology and Parasitology, Medical School, University of Antioquia, Antioquia, Colombia; 3 Nuffield Department of Women's & Reproductive Health, University of Oxford, Women's Centre, Level 3, John Radcliffe Hospital, Oxford; 4 Department of Basic Sciences, College of Medicine, Mohammed Bin Rashid University of Medicine and Health Sciences, Dubai, United Arab Emirates; 5 Division of Medical Physiology, Faculty of Medicine and Health Sciences, Stellenbosch University, Tygerberg, South Africa

Dear Editor,

Although humans have made significant progress in understanding reproductive events, nature still has more information to reveal. Towards the end of the 19 ^th^ century, humans started studying reproductive processes such as gametogenesis, fertilization, and embryo development. 1 Since sperm and egg roles in fertilization were introduced in 1870 for the first time, reproductive biologists tried to emulate the natural reproductive processes despite the significant lack of knowledge regarding *in vivo* reproductive mechanisms. In the quest to mimic or overcome natural reproduction processes, numerous attempts have been made to produce embryos from non-germ cells. 2 3 These trials ultimately culminated in the birth of Dolly the sheep 4 as a result of somatic cell nuclear transfer, starting a new era of cloning or asexual reproduction. Though not fully understood at the time (1959), the work of Chang 5 regarding the fertilization of rabbit ova *in vitro* , paved the way for the application of artificial reproductive techniques (ART) in humans. Ever since then, it has always been considered that the best evidence for these technologies to be accepted is that the progeny derived from ART are capable of reproducing naturally, especially those conceived by *in vitro* fertilization (IVF). Researchers have tried to ensure *in vitro* maturation of the spermatogonial stem cells transplanted in testes, separated into small pieces, and cultured on agarose, which migrated towards the basal membrane and settled on it, as in the *in vivo* process. 6 On the other hand, have reported that after ovary tissue cryopreservation and orthotopic transplantation result in a 76% spontaneous pregnancy live baby rate in 119 human females 13 out 119 of these patients need also *in vitro* maturation of the oocytes, 7 which allows to postulate that the cryopreservation of ovarian tissue could be a promising method to preserve fertility in humans. However, transformation of the experience and data obtained from animals to humans has been failing.

Researchers hope that during these *in vitro* processes, all the cellular events occur in exactly the same fashion as to mimic the *in vivo* scenario, ultimately resulting in new births. Hence, the importance of demonstrating that Dolly could produce offspring through natural mating. Consequently, the potential for humans to produce offspring from gametes generated *in vitro* is exciting, but in some way rather pretentious as it assumes that the events that happen *in vitro* are the same as those that occur *in vivo* . It is undeniable that all these reproductive developments have amazed society. Although there is plenty of literature supporting the possibility, there are still many key questions to be resolved, such as “What minimum number of cells to obtain a blastocyst to obtain a pregnancy, what is the relationship between the number of cells required to obtain a favorable result?”, “In case of cloning how many nuclei are needed to be injected to produce an embryo?” or the efficiency and scalability of such methods to produce enough gametes for assisted conception treatments and will they be safe to use? Undeniably it appears as if Mother Nature is reminding humans that we are still far from replicating the efficiency of natural reproduction. Louise Brown, the first human born through IVF (test-tube baby), is currently 43 years old and has two living children of her own from natural birth. However, more research is required to obtain better success rates and ensure these *in vitro* processes are safe in all aspects. 8

In cattle, procedures involving *in vitro* oocyte maturation and embryo production find it difficult to achieve 40 births from 100 embryos. Furthermore, to create 100 embryos, it is necessary to start with about 400 oocytes 9 ; thus, a 10-fold reduction is experienced: 400 oocytes leading to 40 births. Considering the inefficiency of *in vitro* produced embryos and the response of gametes it is about one-hundredth of that obtained by naturally produced gametes and conditions. 10 An explanation could be associated with the fact that bovine blastocysts obtained *in vitro* have fewer cells than their *in vivo* counterparts. 9 On the other hand, another example, is the need to use thousands of parental cells to obtain few colonies with spermatogenesis inside the testis, although systems should allow the generation of a larger number every day. 3 It would seem very obvious to believe that researchers have been appropriating step by step every event that nature allows them to know. Two works were recently published on the generation of embryo-like structures “the first synthetic embryo" going over the interaction between intergametes: spermatozoa and oocytes. 11 12 The living structures are expected to further a deep understanding of embryogenesis. Finally, once the problem is fully understood, as described earlier (i.e. all events contributing as a unit in its entirety), it can be concluded that nature shows plasticity on a daily basis. In the end, nature will reveal its answers progressively as knowledge translates into understanding. Ultimately it is almost as if we were to engage in dialogue with Mother Nature and asked her to reveal her secrets based on our knowledge and understanding of live births, matured eggs, spermatozoa, and oocytes potentially derived from different cell types. However, nature always has the upper hand and it is as if she would respond with: "Only when you are capable of matching some of my processes, I will let you in on the next secret step. However, it is clear that there is still a long way to go because your way of thinking possibly is not correct. Once you understand all the possibilities, you will increase the efficiency of each process you are interested in”. The advances in knowledge and control of reproductive events during the preceding 100 years are undeniable, but to try and mimic nature and expect similar results and outcomes we have to make a fundamental paradigm shift in how we approach the problem at hand. When it comes to interventions and reproduction processes in humans and other species, acceptable milestones have been achieved but that does not imply that all are acceptable and good.
